# ﻿Mountainous millipedes in Vietnam. III. Two new dragon millipedes from limestone mountains in northern Vietnam (Polydesmida, Paradoxosomatidae, *Hylomus*), with an identification key to Vietnamese *Hylomus* species

**DOI:** 10.3897/zookeys.1223.139649

**Published:** 2025-01-09

**Authors:** Anh D. Nguyen, Tam T. T. Vu, Thu-Anh T. Nguyen

**Affiliations:** 1 Institute of Ecology and Biological Resources, Vietnam Academy of Science and Technology, 18, Hoangquocviet Rd., Caugiay District, Hanoi, Vietnam Institute of Ecology and Biological Resources, Vietnam Academy of Science and Technology Hanoi Vietnam; 2 Graduate University of Science and Technology, Vietnam Academy of Science and Technology, 18, Hoangquocviet Rd., Caugiay District, Hanoi, Vietnam Graduate University of Science and Technology, Vietnam Academy of Science and Technology Hanoi Vietnam

**Keywords:** Biodiversity, COI barcode, mountainous fauna, Southeast Asia, taxonomy

## Abstract

Two new species of the dragon millipede genus *Hylomus* Cook & Loomis, 1924 are described from mountainous areas in northern Vietnam, namely *Hylomuspiccolo***sp. nov.** and *Hylomusborealis***sp. nov.** The COI barcodes are provided for these species, and an identification key is presented to all Vietnamese *Hylomus* species.

## ﻿Introduction

The term “dragon millipedes” refers to species from seven genera: *Desmoxytes* Chamberlin, 1923; *Hylomus* Cook & Loomis, 1924; *Gigaxytes* Srisonchai, Enghoff & Panha, 2018; *Nagaxytes* Srisonchai, Enghoff & Panha, 2018; *Spinaxytes* Srisonchai, Enghoff & Panha, 2018; *Burmaxytes* Srisonchai, Lin & Panha, 2020 and *Siamaxytes* Srisonchai & Panha, 2024 (Srisonchai et al. 2018a, 2018b, 2018c, 2018d, 2020, 2024). While *Burmaxytes* is currently exclusively known in Myanmar, four of these genera — *Gigaxytes*, *Nagaxytes*, *Spinaxytes*, and *Siamaxytes* — are endemic to Thailand; and *Desmoxytes* is known from both Thailand and Malaysia (Srisonchai et al. 2018a). The remaining genus, *Hylomus*, comprises 38 species distributed in southern China (19 species), Vietnam (16), Laos (3), and Thailand (1) (Srisonchai et al. 2018a, Golovatch 2019, Nguyen and Sierwald 2019, Nguyen et al. 2019).

In Vietnam, *Hylomus* is the only known group of dragon millipedes, with 16 species described to date, including three considered troglobiotic (Attems 1938, 1953; Golovatch 2019; Nguyen et al. 2019, 2021). All species, except the post 2018 described species, were originally placed in *Desmoxytes* but have recently been reallocated under *Hylomus* (Srisonchai et al. 2018a). The Vietnamese species are listed in alphabetical order below.

1. *Hylomusasper* (Attems, 1937) from Da Nang and Ninh Thuan provinces.
2. *Hylomuscattienensis* (Nguyen, Golovatch & Anichkin, 2005) from Cat Tien National Park, Dong Nai Province.
3. *Hylomuscervarius* (Attems, 1953) from Sa Pa, Lao Cai Province.
4. *Hylomusenghoffi* (Nguyen, Golovatch & Anichkin, 2005) from Phong Nha – Ke Bang National Park, Dong Nai Province.
5. *Hylomusgrandis* (Golovatch, VandenSpiegel & Semenyuk, 2016) from Kon Chu Rang Nature Reserve, Gia Lai Province.
6. *Hylomushostilis* (Golovatch & Enghoff, 1994) from Tam Dao National Park, Vinh Phuc Province.
7. *Hylomusnamek* Nguyen, Nguyen, Nguyen & Phung, 2019 from Duc Xuan commune, Ha Giang Province.
8. *Hylomuspilosus* (Attems, 1937) from Cat Tien National Park, Dong Nai Province and Hon Ba Mountain, Khanh Hoa Province.
9. *Hylomuspropinquus* Golovatch, 2019, troglobiotic, from Ba Be National Park, Bac Kan Province.
10. *Hylomusproximus* (Nguyen, Golovatch & Anichkin, 2005) from Van Ban commune, Lao Cai Province.
11. *Hylomussaiyans* Nguyen, Nguyen, Nguyen & Phung, 2019 from Cuc Phuong National Park, Ninh Binh Province and Tam Dao NP, Vinh Phuc Province.
12. *Hylomussolenophorus* Nguyen, Nguyen & Eguchi, 2021 from Hoang Lien National Park, Lao Cai Province.
13. *Hylomussongoku* Nguyen, Nguyen, Nguyen & Phung, 2019, troglobiotic, from Xuan Son National Park, Phu Tho Province.
14. *Hylomusspecialis* (Nguyen, Golovatch & Anichkin, 2005) from Ngoc Linh Mountain, Kon Tum Province.
15. *Hylomusspectabilis* (Attems, 1937) from Ba Na – Nui Chua National Park, Da Nang City.
16. *Hylomussrisonchai* Golovatch, 2019, troglobiotic, from Tra Linh District, Cao Bang Province.

This work is devoted to a better understanding of *Hylomus* diversity through the descriptions of two new species from limestone areas of northern Vietnam.

## ﻿Material and methods

Millipede specimens were collected from limestone areas in northern Vietnam during expeditions organized by the Institute of Ecology and Biological Resources (IEBR), Vietnam Academy of Science and Technology.

Morphological characters were investigated under an Olympus SZX16 stereomicroscope. Gonopods were dissected for morphological examination and photographed. Color images were taken at various focal planes using a Sony A6000 camera coupled to a SMZ800N Nikon stereomicroscope. UV images were taken using the aforementioned camera-microscope in combination with illumination from a Nichia Convoy UV flashlight. Images were then stacked using Helicon Focus version 7.0 and assembled in Adobe Photoshop CS6.

For the scanning electron microscope (SEM), gonopods were dissected from the body, dehydrated using a series of ethanol concentrations, 90%, 95%, and 99%, for 24 hours, mounted on an aluminum stub, and then sputter coated with gold. SEM images were taken using the Prisma E system (ThermoFisher Scientific) at IEBR.

Total genomic DNA was extracted from the legs of midbody rings using a Qiagen DNeasy Blood and Tissue Kit. A 680 base pairs fragment of the mitochondrial gene, cytochrome *c* oxidase subunit I (COI), was amplified using a pair of universal primers, LCO1490 and HCO2198 (Folmer et al. 1994). Polymerase chain reaction (PCR) conditions for amplification of the COI gene follow those of Nguyen et al. (2017) as follows: an initial denaturation at 95 °C for 2 min followed by 36 cycles of 95 °C for 20 s, 42 °C for 45 s and 72 °C for 1 min, and a final extension at 72 °C for 5 min. The successfully amplified PCR products were submitted to GenLab (Vietnam) for purification and sequencing. COI sequences were checked and confirmed using BLASTN 2.6.0+ search (Zhang et al. 2000) and registered into GenBank with accession numbers.

Holotypes and paratypes are deposited in the myriapod collection, Institute of Ecology and Biological Resources (IEBR).

### ﻿Abbreviation

**IEBR-Myr** Institute of Ecology and Biological Resources, Myriapod collection.

## ﻿Results

### ﻿Taxonomy

**Class Diplopoda de Blainville in Gervais, 1844**

**Order Polydesmida Pocock, 1887**

**Family Paradoxosomatidae Daday, 1889**

**Genus *Hylomus* Cook & Loomis, 1924**

#### ﻿ Hylomus piccolo sp. nov.

Taxon classificationAnimaliaPolydesmidaHylomus

831524D8-E34E-5890-B862-2D92AB730445

https://zoobank.org/7B27F484-2BBB-47DF-81E4-86A671ED853E

Figs 1 , 2 , 3 , 4

##### Material examined.

***Holotype*.** Vietnam • 1 male; Cao Bang Province, Pia Oac - Pia Den National Park; 22.5943°N, 105.8846°E; 1200 m a.s.l.; 9 May 2021; Anh D. Nguyen leg.; bushes; IEBR-Myr 904H. ***Paratypes*.** Vietnam • 10 males, 5 females; same data as for holotype; EBR-Myr 904P • 1 male, 1 female; same data as for holotype; IEBR-Myr 901.

##### Diagnosis.

The species can be discriminated from the congeners by the presence of long spiniform paraterga, midbody metaterga with only two rows of 1+1 small setiferous spines in the middle and 2+2 longer setiferous spines near posterior margin; male femora 6 with a large tubercle ventrally; sternite 5 with a large, sparsely setose, bifid, trapeziform lamina between male coxae 4; epiproct without conspicuous setiferous knobs near tip; gonopod lamina lateralis broadly rounded, partly folded to sheathe distal part of solenomere; gonopod lamina medialis with a small rounded lobe at middle.

The new species is similar to *Hylomusnamek*Nguyen et al., 2019 but differs from this species by the following characters: the ventral side of male femora 6 with a big, robust tubercle in the middle (vs. femora 6 and 7 with large tubercles); longer spiniform paraterga; and gonopod lamina medialis with a small rounded lobe in the middle (vs. without processes).

##### Etymology.

The name refers to “*piccolo*”, a main character of the Japanese manga “Dragon balls” by Toriyama Akira (Japan). Noun in apposition.

##### Description.

Length c. 11.6–12.8 mm (male), 13.9–15.2 mm (female); width of midbody pro- and metazona (distance between two paratergal tips) 0.6–0.8 mm (male), 1.12–1.25 mm (female) and 3.1–3.3 mm (male), 3.4–3.6 mm (female), respectively. Holotype length c. 11.8 mm, width of midbody pro- and metazona 0.74 mm and 3.2 mm, respectively.

***Coloration***: Generally darkish-red to darkish-brown except paraterga, sterna, legs whitish-yellow; distal part of main branch pinkish.

**Figure 1:**
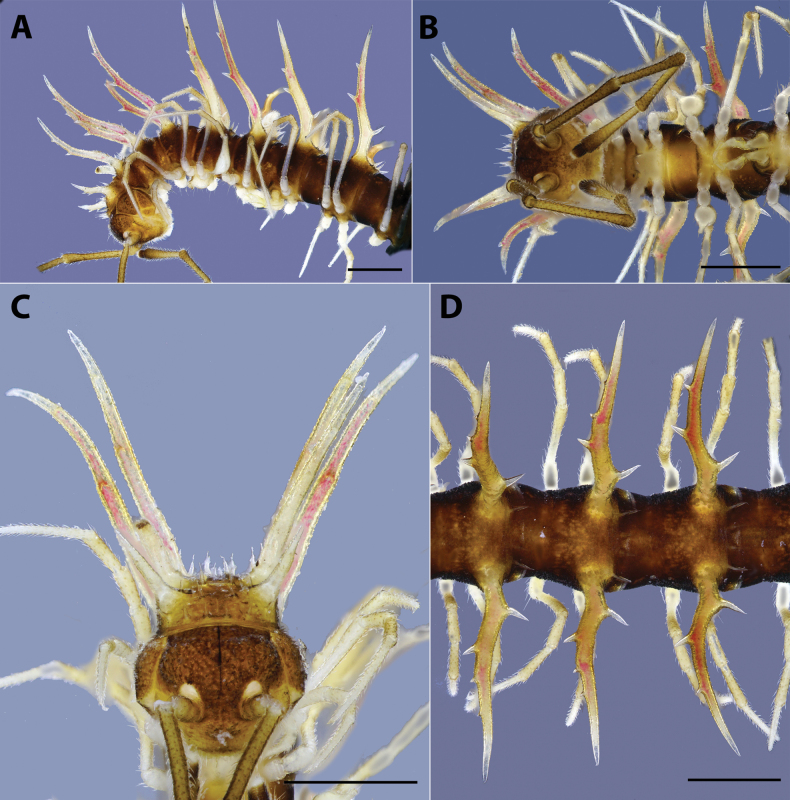
. *Hylomuspiccolo* sp. nov., holotype (IEBR-Myr 904H) **A, B** anterior-most body part, lateral view (**A**), ventral view (**B**) **C** head, anterior view **D** body rings 8–10, dorsal view. Scale bars: 1 mm.

***Head*** (Fig. 1A–C): Clypeolabral region densely setose, vertex sparsely setose. Epicranial suture distinct, dividing frons into two equal parts; with setae along the suture. Antenna slender, extremely long, reaching back to body ring 7 if stretched along the body axis; antennomere 1 < 7 < 6 < 2 < 3 = 4 = 5 in length.

***Collum*** (Fig. 1C): Slightly narrower than head; surface dull, coarsely microgranulate; with two rows of spines: 3+3 spines in anterior row and 2+2 spines in posterior row. Paraterga of collum well developed, spiniform; directed dorsad; highly elevated above dorsal surface; with two conspicuous teeth on anterior side.

**Figure 2.**
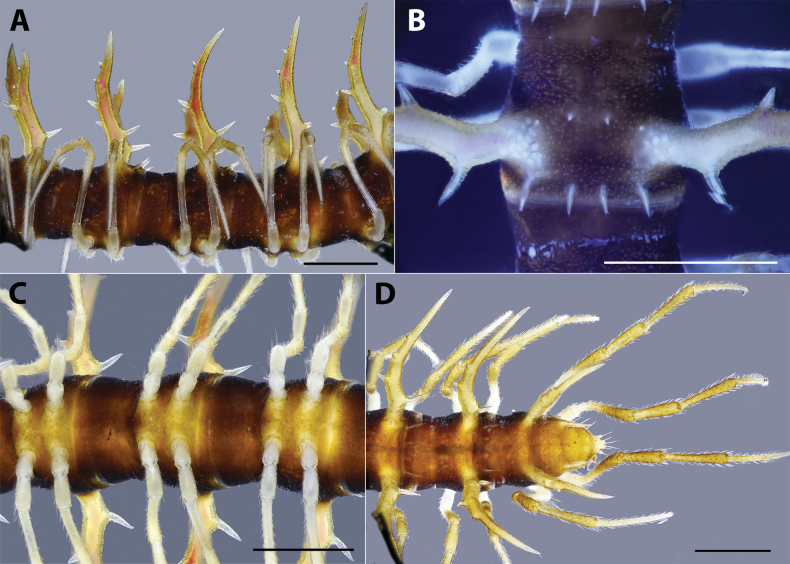
*Hylomuspiccolo* sp. nov., holotype (IEBR-Myr 904H) **A** body rings 8–10, lateral view **B** body ring 10, dorsal view (under UV light) **C** body rings 8–10, ventral view **D** posterior-most body part, dorsal view. Scale bars: 1 mm.

***Body rings***: Ring 3 < 4 < 2 = 5–16 in width, thereafter gradually tapering towards telson. Prozona finely shagreened; metazona and pleura with microgranulations. Transverse sulcus present, but inconspicuous on metaterga 5–18. Axial line missing. Metaterga with two rows of 1+1 smaller setiferous spines in middle and 2+2 longer setiferous spines near posterior margin (Fig. 2B). Suture between pro- and metazona broad, very shallow. Pleurosternal carinae present as a complete keel on body rings 2–3, then missing on subsequent body rings.

***Paraterga*** (Figs 1, 2): Very well developed; directed dorsad; long and spiniform with a large branch and 2–3 tiny teeth on anterior side (2 on poreless body rings and 3 on pore-bearing rings) and with 1 larger spine on posterior side. Paratergum on ring 19 directed caudad. Ozopore located between the second tooth and main branch of paraterga, visible in dorsal view.

**Figure 3.**
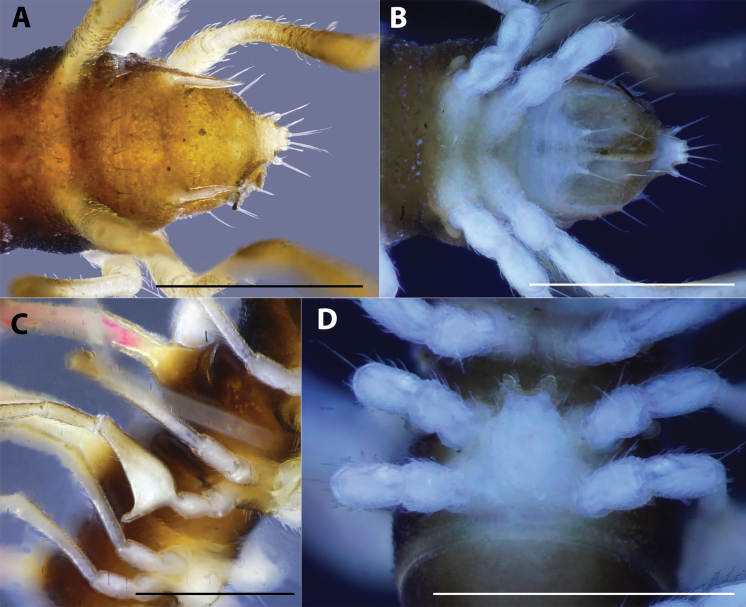
*Hylomuspiccolo* sp. nov., holotype (IEBR-Myr 904H) **A** telson, dorsal view **B** telson, ventral view (under UV light) **C** leg 6 **D** sternum 5, subposterior view (under UV light). Scale bars: 1 mm.

***Telson*** (Fig. 3A, B): Epiproct without conspicuous setiferous knobs near tip, but broadly truncated; tip with four spinnerets; lateral tubercles well developed (Fig. 3A). Hypoproct subtrapeziform, with two distolateral, completely separated, setiferous knobs (Fig. 3B).

***Legs***: Extremely long, slender and thin, c. 1.8–2.0 times as long as midbody height. Prefemora not swollen. Male femora 6 each ventrally with a large, robust tubercle in middle (Figs 1A, 3C).

***Sterna***: With distinct cross-impressions, no modification – except a large, sparsely setose, bifid, trapeziform lamina between male coxae 4 (Figs 1A, 3D).

**Figure 4.**
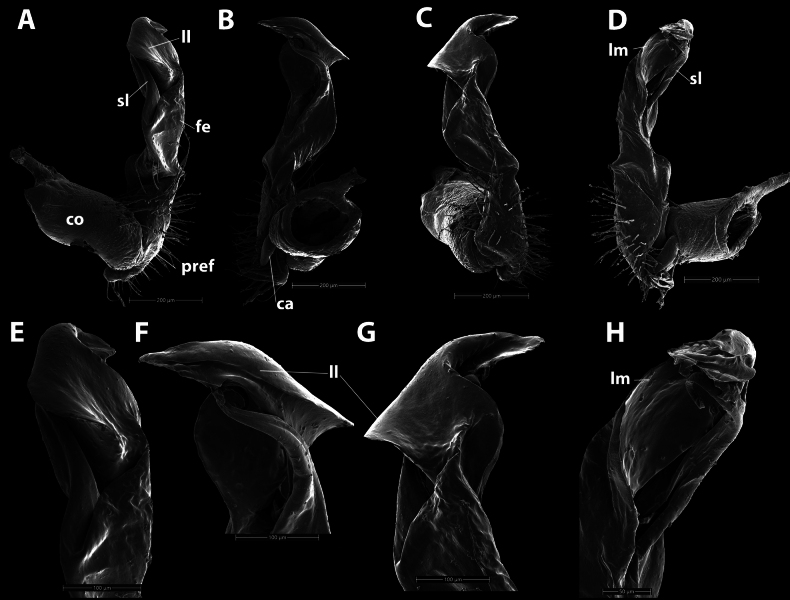
*Hylomuspiccolo* sp. nov., holotype (IEBR-Myr 904H), SEM**A–D** left gonopod, lateral view (**A**), dorsal view (**B**), ventral view (**C**), mesal view (**D**) **E–H** distal part of left gonopod, lateral view (**E**), dorsal view (**F**), ventral view (**G**), mesal view (**H**). Abbreviations: **co** = coxite; **pref** = prefemorite; **fe** = femorite; **ca** = canula; **lm** = lamina medialis; **ll** = lamina lateralis; **sl** = solenomere. Scale bars: 200 µm (**A–D**); 100 µm (**E–G**); 50 µm (**H**).

***Gonopod*** (Fig. 4): Suberect. Coxite (**co**) cylindrical, larger than femorite, sparsely setose distodorsally. Prefemorite (**pref**) densely setose, equal to femorite as well. Femorite (**fe**) slightly enlarged distad (from ventral view); without a demarcation with postfemoral region. Postfemoral region inconspicuous. Solenophore (**sph**) well developed; lamina lateralis (**ll**) broadly rounded, partly folded to sheathe distal part of solenomere; lamina medialis (**lm**) with a small rounded lobe at about midway. Seminal groove running entirely on mesal side, then entering a flagelliform solenomere (**sl**), solenomere partly sheathed by solenophore. Tip of solenophore rounded.

##### DNA barcoding.

(Appendix 1) A fragment of the COI gene is accessioned at NCBI GenBank with the following accession number PQ676351. The new species exhibits a COI gene similarity of 86.22% and 86.57% (OR765910 and MG669370, respectively) identity with *Hylomuscervarius* (Attems, 1953).

#### ﻿ Hylomus borealis sp. nov.

Taxon classificationAnimaliaPolydesmidaHylomus

3B05C4F6-31AD-50F7-B51A-9B5596DE560E

https://zoobank.org/0459DB77-C98E-4DC6-B79F-E13950DAED19

Figs 5 , 6 , 7

##### Material examined.

***Holotype*.** Vietnam • 1 male; Cao Bang Province, Pia Oac - Pia Den National Park, on the way to Hang Ong; 22.5540°N, 105.8622°E; 850m a.s.l.; 8 May 2021; Anh D. Nguyen leg.; bushes; IEBR-Myr 908H. ***Paratypes*.** Vietnam • 2 females; same data as for holotype; IEBR-Myr 908P • 2 females; same data as for holotype; IEBR-Myr 906 • 2 females; same data as for the holotype, but 8 Jun. 2020; IEBR-Myr 851 • 1 female; same data as for sample IEBR-Myr 851; IEBR-Myr 854.

##### Diagnosis.

The species can be discriminated from the congeners by the presence of spiniform paraterga; metaterga densely covered with microgranulations; midbody metaterga with two rows of setiferous spines: 2+2 in anterior row and 2+2 near posterior margin, the anterior row hardly seen, the posterior row more distinct; male femora 6 each with a large tubercle ventrally; sternite 5 with a large, sparsely setose, bifid, trapeziform lamina between male coxae 4; epiproct with several evident setiferous knobs near tip; gonopod solenophore partly folded to sheathe distal part of solenomere; tip of solenophore consisting of seven overlapping laminae.

The new species is similar to *H.proximus* in body size and shape, but the two species are distinguished by the number of metatergal posterior spines (2+2 vs 3+3), male femoral modifications (femur 6 vs femora 5 & 6), and gonopod conformation. The new species has a well-developed gonopod solenophore (sph); a broadly rounded lamina medialis, partly folded to sheathe distal part of solenomere; and gonopod tip consisting of seven overlapping laminae while *H.proximus* has a gonopod femorite that is subequal to the postfemoral region in length; both solenophore and solenomere long; and a serrated solenophore tip.

*Hylomusborealis* sp. nov. is also similar to *H.jeekeli* (Golovatch & Enghoff, 1994) from northern Thailand in terms of general body and gonopod shape. However, the new species can be distinguished from it by the combination of these characters: smaller in size with 10.4 mm in males and 12.3–13.4 mm in females (vs 15–16 mm in males and 18–20 mm in females); metaterga with 2+2 spines in posterior rows (vs 3+3 spines); modification in only femur 6 (vs femora 6 and 7); tip of solenophore consisting of seven overlapping laminae and not serrate (vs serrated solenophore).

##### Etymology.

An adjective epithet “*borealis*” refers to the northern-most province (Cao Bang) of Vietnam, the type locality.

##### Description.

Holotype length 10.4 mm, width of mid pro- and metazona 0.6 mm and 1.8 mm (distance between two paratergal tips), respectively. Female length 12.3–13.4 mm, width of mid pro- and metazona 0.9–1.0 mm and 1.7–1.8 mm, respectively.

***Coloration***: Generally dark to castaneous brown except paratergal bases, sterna, leg coxae and prefemora whitish-yellow.

**Figure 5.**
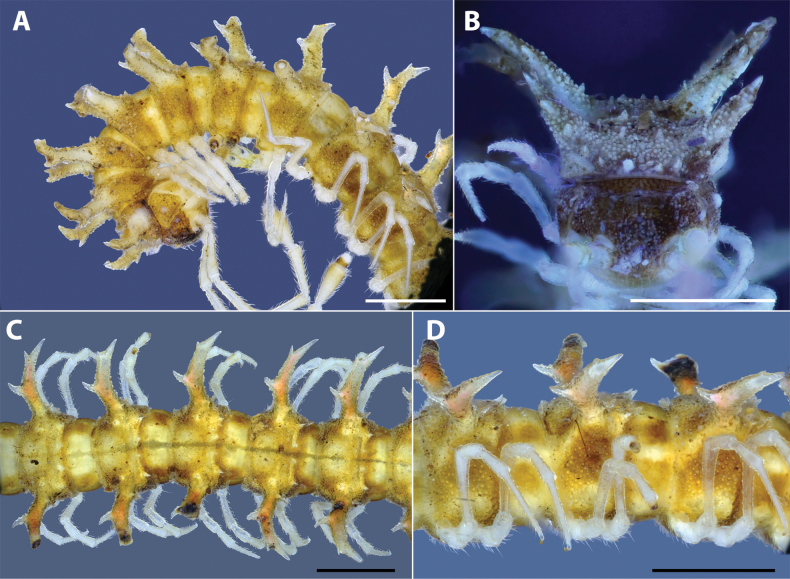
*Hylomusborealis* sp. nov., holotype (IEBR-Myr 908H) **A** anterior-most body part, lateral view **B** frons and collum, anterior view (under UV light) **C** body rings 8–12, dorsal view **D** body rings 8–10, lateral view. Scale bars: 1 mm.

***Head*** (Fig. 5A, B): Clypeolabral region densely setose, vertex sparsely setose. Epicranial suture distinct, dividing frons into two equal parts; with setae along suture. Antenna slender, extremely long, reaching to body ring 5 if stretched along the body axis; antennomere 1 < 7 < 6 < 2 < 3 = 4 = 5 in length.

***Collum*** (Fig. 5B): Subequal to head in width; surface dull, coarsely and densely microgranulate, with three rows of spines: 3+3 spines in anterior row, 1+1 spines in intermediate row, and 2+2 spines in posterior row; all spines equal in size. Paratergum well developed; directed dorsad; highly elevated above dorsal surface; with two conspicuous teeth on anterior side.

**Figure 6.**
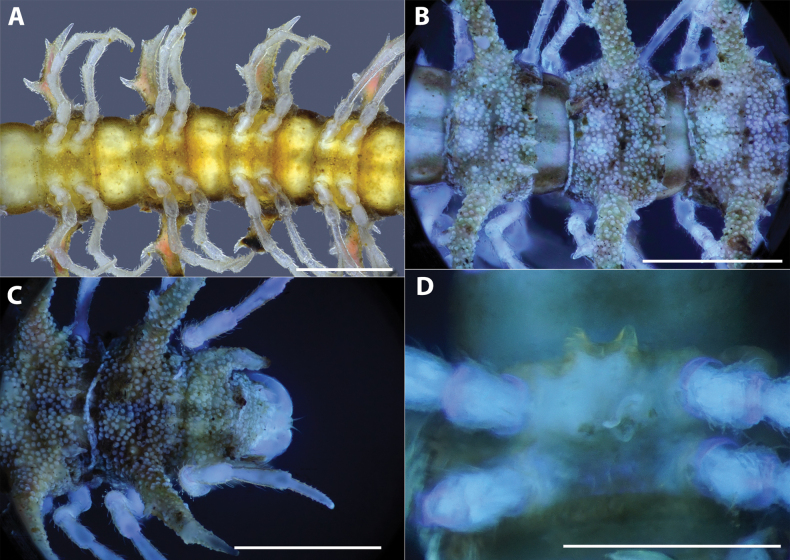
*Hylomusborealis* sp. nov., holotype (IEBR-Myr 908H) **A** body rings 8–10, ventral view **B** body rings 8–10, dorsal view (under UV light) **C** telson, dorsal view (under UV light) **D** sternum 5, subposterior view. Scale bars: 1 mm.

***Body rings***: Rings 3 < 4 < 2 = 5–16 in width, thereafter gradually tapering towards telson. Prozona finely shagreened; metazona and pleura with microgranulations. Transverse sulcus present, but inconspicuous on metaterga 5–18. Axial line missing. Metaterga with two rows of setiferous spines: 2+2 spines in anterior row and 2+2 spines near posterior margin (Figs 5C, 6B), the anterior row hardly visible, the posterior row more distinct (Fig. 6B). Suture between pro- and metazona broad, very shallow. Pleurosternal carinae present as a complete keel on body rings 2–3, then missing on subsequent body rings.

***Paraterga*** (Fig. 5): Very well developed; directed laterodorsad; antler-shaped with a large branch and 2 small teeth on anterior side and 1 smaller spine on posterior side. Ozopore located between the first tooth and main branch of paraterga, visible in dorsal view.

***Telson*** (Fig. 6C): Epiproct with several evident setiferous knobs near tip; tip with four spinnerets; lateral tubercles well developed. Hypoproct sub-trapeziform, with two distolateral, completely separated, setiferous knobs.

***Legs***: Extremely long, slender and thin, c. 1.5–1.6 times as long as midbody height. Prefemora not swollen. Male femora 6 each ventrally with a large, robust tubercle in middle.

***Sterna***: with distinct cross-impression, no modification – except a large, sparsely setose, trapeziform lamina carrying two distal, separated lobes between male coxae 4 (Fig. 6D).

**Figure 7.**
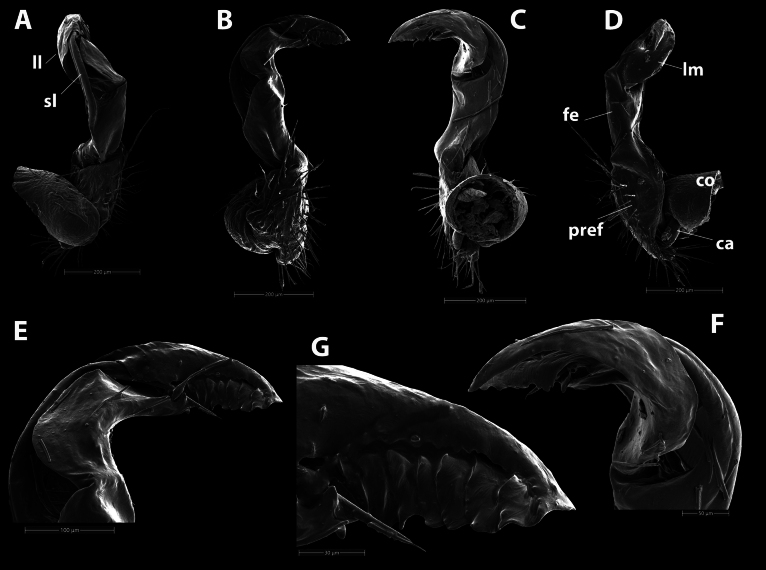
*Hylomusborealis* sp. nov., holotype (IEBR-Myr 908H), SEM**A–D** left gonopod, lateral view (**A**), ventral view (**B**), dorsal view (**C**), mesal view (**D**) **E–G** distal part of left gonopod, ventral view (**E**), dorsal view (**F**) **G** tip of left gonopod, ventral view. Abbreviations: **co** = coxite; **pref** = prefemorite; **fe** = femorite; **ca** = canula; **lm** = lamina medialis; **ll** = lamina lateralis; **sl** = solenomere. Scale bars: 200 µm (**A–D**); 100 µm (**E**), 50 µm (**F**), 30 µm (**G**).

***Gonopod*** (Fig. 7): Suberect. Coxite (**co**) cylindrical, much shorter than femorite, sparsely setose distodorsally. Prefemorite (**pref**) densely setose, shorter than femorite as well. Femorite (**fe**) slightly enlarged distad, without a demarcation with postfemoral region. Postfemoral region slightly twisted mesad. Solenophore (**sph**) well developed; lamina medialis (**lm**) broadly rounded, partly folded to sheathe distal part of solenomere, lamina lateralis (**ll**) well developed. Seminal groove running entirely on mesal side, then entering a flagelliform solenomere (**sl**) which is partly sheathed by solenophore. Tip of gonopod consisting of seven overlapping laminae (Fig. 7G).

##### DNA barcoding.

(Appendix 1) A fragment of the COI gene is accessioned at NCBI GenBank with the following accession number PQ676352. The new species exhibits a COI gene similarity of 85.29% identity with *Hylomusproximus* Nguyen, Golovatch & Anichkin, 2005 (MG669371) and 83.11% identity with *Desmoxytestakensis* Srisonchai, Enghoff, Likhitrakarn & Panha, 2016 (OR765894).

### ﻿Key to *Hylomus* species in Vietnam

(based on male characters)

**Table:** 

1	Body unpigmented. Troglobiotic	**2**
–	Body pigmented. Non-troglobiotic	**4**
2	Paraterga well developed, spiniform. Metaterga with 56 + 56 setiferous spines in posterior row of midbody ring. Lamina lateralis of gonopod solenophore with dense setal region	***H.songoku***
–	Paraterga small, poorly developed. Metaterga with less than 4 + 4 setiferous knobs in posterior row of midbody ring. Lamina lateralis of gonopod solenophore without dense setal region	**3**
3	Metaterga with 2 + 2 setiferous knobs in posterior row of midbody ring. Gonopod lamina lateralis with a small, but evident, slender, slightly twisted, apicomesal process, about half as long as a slender, distally conspicuously rugged and denticulate lamina medialis	***H.srisonchai***
–	Metaterga with 3–4 + 3–4 setiferous knobs in posterior row of midbody ring. Gonopod lamina lateralis a simple rounded lobe, while lamina medialis deeply split into a long distal spine and a strongly bifid basal ribbon, both slightly spinulate	***H.propinquus***
4	Paraterga wing-shaped	***H.spectabilis***
–	Paraterga antler- or spine-shaped	**5**
5	Paraterga antler-shaped	**6**
–	Paraterga spine-shaped	**15**
6	Epiproct without conspicuous setiferous knobs near tip	**7**
–	Epiproct with several evident setiferous knobs near tip	**12**
7	Metaterga smooth, more or less shining. Male femora 5–9 unmodified	***H.asper***
–	Metaterga rough, dull, granular. Male femora 5/6/7/9 modified, inflated	**8**
8	Metaterga with 3 + 3 setiferous spines in posterior row. Male femora 6, 7 and 9 humped ventrally. Fifth sternum with a pair of tubercles between male coxae 4	***H.cervarius***
–	Metaterga with 2 + 2 setiferous spines in posterior row. Male femora 6 and 7, sometimes femur 5 modified, each with a large hump on ventral side. Fifth sternum with either a rectangular process or a bifid, trapeziform lamina between male coxae 4	**9**
9	Body light or darkish-brown. Fifth sternum with a bifid, trapeziform lamina between male coxae 4	**10**
–	Body reddish or pinkish. Fifth sternum with four round tubercles on a prominent, elevated, rectangular lamina between male coxae 4	***H.enghoffi***
10	Only male femur 6 with a big, robust ventral tubercle in middle	***H.piccolo* sp. nov.**
–	Male femora 6, 7, sometimes 5 humped ventrally	**11**
11	Male femora 6, 7 humped ventrally. Tip of solenophore lobuliform, broadly rounded	***H.namek***
–	Male femora 6, 7, sometimes 5, humped ventrally. Tip of solenophore acute	***H.saiyans***
12	Metaterga with at least 3 + 3 spines in posterior row	**13**
–	Metaterga with only 1 + 1 setae in posterior row	***H.cattienensis***
13	Body pink to red. Metaterga with numerous microsetae, and 4 + 4 spines in posterior row	***H.pilosus***
–	Body dark to castaneous brown. Metaterga with microganulations, and with less 4+4 spines in posterior row	**14**
14	Metaterga with 3 + 3 spines in posterior row. Male femora 5 and 6 with a large ventral tubercle	***H.proximus***
–	Metaterga with 2 + 2 spines in posterior row. Male femur 6 with a large ventral hump	***H.borealis* sp. nov.**
15	Metaterga with 1 + 1 spines in posterior row. Gonopod subfalcate, femorite slightly curved; solenophore long	***H.specialis***
–	Metaterga with 2 + 2 tubercles/spines in posterior row. Gonopod falcate; solenophore short, pointed terminally	**16**
16	Fifth sternum with two setiferous tubercles between male coxae 4, clearly separated. Antenna long and slender	***H.grandis***
–	Fifth sternum with a bifid setiferous trapeziform lamina between male coxae 4. Antenna short and stout	***H.hostilis***

## ﻿Discussion

The genus *Hylomus* is widely distributed from southern China to northern Thailand, all parts of Laos, and all regions of Vietnam, with Srisonchai et al. (2024) suggesting that the Tenasserim mountain range, located between Thailand and Myanmar, may serve as the center of origin for dragon millipedes, including another six genera. While *Hylomus* appears to be primarily confined to the north and south Annamite Mountain ranges as indicated by Srisonchai et al. (2024), a comprehensive phylogenetic analysis of the genus remains lacking. Preliminary studies, such as those by Nguyen et al. (2019), which used a short fragment of the 16S rRNA gene, and Srisonchai et al. (2024), which conducted a broader phylogenetic assessment of dragon millipedes, have yielded different results: *Hylomus* was found to be monophyletic in Nguyen et al. (2019) but non-monophyletic in Srisonchai et al. (2024). Additionally, most *Hylomus* species exhibit a diverse range of paratergal morphologies, including winglike (*H.spectabilis*), spinelike (*H.hostilis*), subspiniform (*H.piccolo*), and antlerlike (*H.pilosus*) forms, as categorized by Golovatch and Enghoff (1994) and Srisonchai et al. (2024). The evolutionary relationships and diversification of paratergal morphology within *Hylomus* remain unclear, highlighting the need for more comprehensive phylogenetic studies using additional gene markers.

With an area exceeding 60,000 km^2^, Vietnam is renowned for its rich karst ecosystem, and is heavily reliant on its limestone karst regions, known for their extraordinary species diversity (Tuyet 2001). Composed primarily of calcium carbonate, these karsts have developed over millions of years, hosting a wide variety of plant and animal species. According to Tuyet (2001), the phylum Chordata alone includes 541 species across 80 families, 40 orders, and 5 classes, with several rare and endangered species. Recent discoveries of new millipede species, such as *Paracortinakyrang* (Nguyen et al., 2023), *Hyleoglomerishalang* (Kuroda et al., 2022), *Hyleoglomerisalba* (Kuroda et al., 2022), and *Pacidesmustuachua* (Nguyen et al., 2024), in limestone caves further highlight the rich yet underexplored biodiversity of the region. Positioned at the center of diversity for *Hylomus*, Vietnam may contain many undiscovered species, awaiting identification through comprehensive surveys.

## ﻿Conclusion

The number of *Hylomus* species in Vietnam has increased to 16, with the addition of two new species, *H.piccolo* sp. nov. and *H.borealis* sp. nov., from limestone forests in northern Vietnam. More extensive surveys and additional DNA data are needed to fully clarify the diversity, biogeography and phylogenetics of the genus *Hylomus* in Vietnam and Southeast Asia.

## Supplementary Material

XML Treatment for Hylomus piccolo

XML Treatment for Hylomus borealis

## ﻿Additional information

### Author ORCIDs

Anh D. Nguyen https://orcid.org/0000-0001-9273-0040

Tam T. T. Vu https://orcid.org/0000-0003-1145-975X

Thu-Anh T. Nguyen https://orcid.org/0000-0002-0564-3051

### Data availability

All of the data that support the findings of this study are available in the main text.

## ﻿Appendix 1

>IEBR_Myr_904 [organism = Hylomuspiccolo sp.nov.]

TCTTCATTAAGAGGGATTATTCGGGTTGAGTTAAGAGGGTCTGGTAGATTAATTGGGGATGACCAG ATTTACAATGTAATAGTTACTGCTCATGCTTTTGTTATAATTTTTTTTATGGTTATGCCTATTA TAATCGGGGGGTTTGGAAATTGGTTGGTTCCTATTATAATTGGAGCTCCTGATATAGCTTTTCCT CGGATAAATAATTTAAGTTTTTGGTTACTACCTCCTTCTTTTCTTTTATTAATGATATCTTCTATG GTGGAAGGGGGAGTTGGAACAGGATGAACAGTTTATCCTCCTTTAGCTTCAAGTTTATTCCATGAG GGGTCAGCTGTTGATTTGGCTATTTTTTCTTTACATTTGGCTGGGGCCTCTTCTATTTTGGGTGCT

>IEBR_Myr_908 [organism = Hylomusborealis sp.nov.]

TCTTCTTTAAGAGGGATTATTCGGGTTGAATTAAGAGGGACTGGAAGATTAATCGGTGATGACC AGATTTATAATGTTATGGTAACAGCTCATGCTTTTGTAATGATTTTTTTTATGGTTATGCCT ATTATAATCGGGGGGTTCGGGAATTGATTGGTGCCTATTATGATTGGGGCTCCTGACATGGCT TTTCCGCGGATAAATAATTTGAGTTTTTGATTGTTACCTCCTTCTTTTTTTTTATTGATGTTGT CGTCAATGGTGGAGGGGGGGGTAGGGACTGGATGGACAGTTTATCCTCCGCTGGCTTCTAGATT GTTTCATGAGGGTTCGGCTGTGGATTTGGCTATTTTTTCTTTACATTTAGCTGGGGCTTCCTCTA TTTTAGGGGCTATTAATTTTATTTCTACTGTTATCAATATGCGGGTTCGTGGAATAATTTTTGAG CGTATTCCACTGGTTGTTTGATCGGTTTTTATTACTGCTATTCTGTTATTGTTATCTTTGCCTG TTCTGGCTGGGGCTATTACAATGTTATTAACTGATCGGAACTTTAATACAACTTTTTTTGACCC GGCTGGAGGGGGGGA
